# Cardiac Arrest in an Adolescent with Pulmonary Embolism

**DOI:** 10.21980/J8135T

**Published:** 2021-10-15

**Authors:** Matthew Myers, Courtney Devlin

**Affiliations:** * University of Pittsburgh Medical Center Harrisburg, Department of Emergency Medicine, Harrisburg, PA

## Abstract

**Audience:**

The target audience of this simulation is emergency medicine residents and medical students. The simulation is based on a real case of a 13-year-old female who presented with seizures and hypoxia and was ultimately diagnosed with pulmonary embolism. The case highlights diagnosis and management of an adolescent with new onset seizures, deterioration in status, and treatment options in pediatric cardiac arrest due to pulmonary embolism.

**Background:**

Pulmonary embolism (PE) is an uncommon cause of hypoxia in children making diagnosis difficult. A study looking at 23 years of admission and autopsy data on children found the incidence of clinically significant pulmonary embolism to be 25 per 100,000.^1^ However, when children present to the emergency department with hypoxia and altered mental status, a diagnosis of pulmonary embolism cannot be excluded. Risk factors such as use of estrogen containing oral contraceptives, immobilization, and recent surgery should raise suspicion for pulmonary embolism in the clinically deteriorating adolescent patient.^2^,^3^

**Educational Objectives:**

By the end of the simulation, learners will be able to:

1) develop a differential diagnosis for an adolescent presenting with hypoxia and seizure-like activity
2) discuss the utility of bedside ultrasound in helping to differentiate causes of hypoxia
3) discuss management of cardiac arrest due to PE in the pediatric patient

Secondary learning objectives include:

4) discuss indications for emergent use of thrombolytics and Extracorporeal Membrane Oxygenation (ECMO) while becoming aware of institution-based limitations
5) demonstrate interpersonal communication with family, nursing, pharmacy, and consultants during high stress situations.

**Educational Methods:**

This is a high-fidelity simulation that allows learners to manage the diagnosis and treatment of pulmonary embolism in an adolescent patient. Participants participated in a debriefing after the simulation.

**Research Methods:**The effectiveness of this case was evaluated by surveys given to learners after debriefing. Learners gave quantitative and qualitative results of their feedback using a 1–5 rating scale and leaving written feedback. This case was performed with residents in their first and second years of training.

**Results:**

Feedback was overall positive, with many of the residents giving the case high scores on effectiveness of the simulation in their education. They enjoyed the case and reported they would feel more comfortable in a comparable situation in the future.

**Discussion:**

Pulmonary embolism is an uncommon but important diagnosis for emergency medicine physicians to consider in pediatric cardiac arrest. This case has multiple parts and was based on a real case in our emergency department in which a patient presenting with new seizure-like activity followed by cardiac arrest was ultimately diagnosed with a PE. The case was well received by our learners who felt it improved their identification of this diagnosis and its management.

**Topics:**

Pulmonary embolism, oral contraceptives, altered mental status, pediatric, adolescent, cardiac arrest, ECMO, thrombolytic, hypoxia, emergency medicine, medical simulation.

## USER GUIDE

**Table:** 

List of Resources:	
Abstract	112
User Guide	113
Instructor Materials	116
Operator Materials	127
Debriefing and Evaluation Pearls	131
Simulation Assessment	133

**Learner Audience:**

Emergency Medicine junior residents and senior residents

**Time Required for Implementation:**

Instructor Preparation: 20 minutes

Time for case: 20 minutes

Time for debriefing: 20–25 minutes

**Recommended Number of Learners per Instructor:**

3–4

**Topics:**

Pulmonary embolism, oral contraceptives, altered mental status, pediatric, adolescent, cardiac arrest, ECMO, thrombolytic, hypoxia, emergency medicine, medical simulation.

**Objectives:**

By the end of this simulation session, the learner will be able to:

1. develop a differential diagnosis for an adolescent presenting with hypoxia and seizure-like activity
2. discuss the utility of bedside ultrasound in helping to differentiate causes of hypoxia
3. discuss management of cardiac arrest due to PE in the pediatric patient
4. discuss indications for emergent use of thrombolytics and Extracorporeal Membrane Oxygenation (ECMO) while becoming aware of institution-based limitations
5. demonstrate interpersonal communication with family, nursing, pharmacy, and consultants during high stress situations.

### Linked objectives and methods

Adolescent patients that present to the emergency department with seizure and hypoxia have a wide differential diagnosis. This patient presents with a pulmonary embolism resulting in hypoxia causing a seizure. Pulmonary embolism is typically low on the differential for adolescent patients, but some begin to take oral contraceptives soon after puberty. Learners will first be presented with a critically ill patient and need to rapidly stabilize and form a wide list of differential diagnoses (Objective 1). The patient’s condition then again deteriorates, and the learners will need to complete a repeat primary survey as well as review PALS algorithms to treat cardiac arrest (Objective 3). During the resuscitation phase when learners are reviewing reversable causes of PEA (Pulseless Electrical Activity) they will need to use bedside ultrasound that will demonstrate right heart strain from a pulmonary embolism (Objective 2). To provide proper treatment, learners will have to administer thrombolytics to achieve return of spontaneous circulation (Objective 4). Learners should also discuss ECMO if it is available at their institution (Objective 4). Throughout this simulation, a family member is present and will require frequent updates and support, and learners will need to communicate with family as well as support staff and consultants (Objective 5). At the end of the simulation, learners can discuss the underlying pathophysiology of pulmonary embolism as well as their institutions’ capabilities for ECMO and the closest ECMO centers (Objective 4).

### Recommended pre-reading for instructor

- American Heart Association PALS Course. At: https://cpr.heart.org/en/courses/pals-course-options
- Kline JA. Chapter 56: Venous Thromboembolism Including Pulmonary Embolism. Tintinalli JE, Ma OJ, Yealy DM, eds. *Tintinalli’s Emergency Medicine: A Comprehensive Study Guide*. 9th ed. New York, N.Y: McGraw-Hill Education LLC, 2017. Print.
- Rali P, Gandhi V, Malik K. Pulmonary Embolism. *Crit Care Nurs Q*. 2016; Apr–Jun;39(2):131–8. PMID: 26919674. doi: 10.1097/CNQ.0000000000000106
- Advanced Management Options for Massive and Submassive Pulmonary Embolism. *US Cardiology Review*. 2016;10(1):30–5. doi: https://doi.org/10.15420/usc.2016.10.1.30

### Results and tips for successful implementation

The pilot session of this simulation was performed with emergency medicine residents in their first and second years of training. The effectiveness of the case was measured using a survey completed after the debrief. After the simulation, participants were given a survey rating their understanding of pediatric cardiac arrest prior to and after the simulation lab. They were also to give qualitative feedback on how they felt the simulation went in a comments section at the end of the survey. A scale of 1–5 (where 1 is completely disagree to 5 is completely agree) was used to answer four questions as follows:

1. My knowledge of managing a pediatric patient with cardiac arrest was at my proper level of Post Graduate Year (PGY) training.
2. My knowledge after the simulation lab made me more prepared to manage a pediatric patient suffering from cardiac arrest due to PE.
3. The simulation was valuable to my clinical practice.
4. After completing this simulation lab, I will change my clinical practice when it comes to rapid assessment of a crashing pediatric patient.

In total, seven residents completed the survey. All surveys were anonymous. The mode score for questions two and three was a 5 (completely agree), whereas the mode scores for questions one and four were 4 and 3. These responses suggest that while our learners felt they had a decent background knowledge of the material, the simulation was valuable to their clinical practice and prepared them to manage a similar case in the future.

Based on the comments, a longer debrief will be in place for the next round of this simulation to allow for more question time and discussion. Also, we added the location of the case and available resources (ECMO, pediatric ICU) to the introduction so our learners could act based on these parameters.

The comments from learners are below:

“Excellent case, I would not change much.”

“More discussion time, please.”

“Having a two-part scenario (seizure to PE arrest) is a good approach to shifting gears in a crashing patient situation.”

“I would appreciate more knowledge of where we were/resources available (ex-PICU availability).”

## Supplementary Information



## Figures and Tables

**Table:** 

#1	Finger Stick Glucose
#2	Arterial Blood Gas
#3	Bedside Ultrasound Showing Right Heart Strain
#4	Chest Radiograph
#5	Electrocardiogram
#6	Basic Metabolic Panel
#7	Complete Blood Count
#8	Troponin
#9	Brain Natriuretic Peptide
#10	Pregnancy Test

**Table:** 

**Vitals:**	Heart Rate (HR) 162	Blood Pressure (BP) 129/57	Respiratory Rate (RR) 40
	Temperature (T) 36.4°C	Oxygen Saturation (O_2_Sat) 72% on room air	

**Table:** 

**Vitals:**	Heart rate (HR) 120	Blood pressure (BP) 90/60	Respiratory rate (RR) 6
	Temperature (T) 36.0°C	Oxygen saturation (O_2_Sat) 90%	

**Table:** 

*Basic metabolic panel (BMP)*	
Sodium	137 mEq/L
Chloride	106 mEq/L
Potassium	3.3 mEq/L
Bicarbonate (HCO_3_)	18 mEq/L
Blood Urea Nitrogen (BUN)	9 mg/dL
Creatine (Cr)	1.03 mg/dL
Glucose	155 mg/dL
Calcium	8.8 mg/dL
Anion Gap	13.0

**Table:** 

*Finger Stick Glucose*	140 mg/dL

**Table:** 

*Complete blood count (CBC)*	
White blood count (WBC)	25.0 ×1000/mm^3^
RBC Count	4.60 M/μL
Hemoglobin (Hgb)	12.4 g/dL
Hematocrit (HCT)	38.8%
Platelet (Plt)	373 K/μL
Neutrophils %	39.0%
Lymphocytes %	54.0%

**Table:** 

*Troponin*	6.4 ng/mL

**Table:** 

*Brain Natriuretic Peptide*	152 pg/mL

**Table:** 

*Serum Pregnancy*	negative

**Table:** 

*Arterial Blood Gas (ABG)*	
Arterial pH	7.10
PaCO2 Arterial	66 mmHg
PaO2 Arterial	63 mmHg
Bicarbonate (HCO_3_)	14 mEq/L
Arterial O2 Sat	51%

**Figure:**
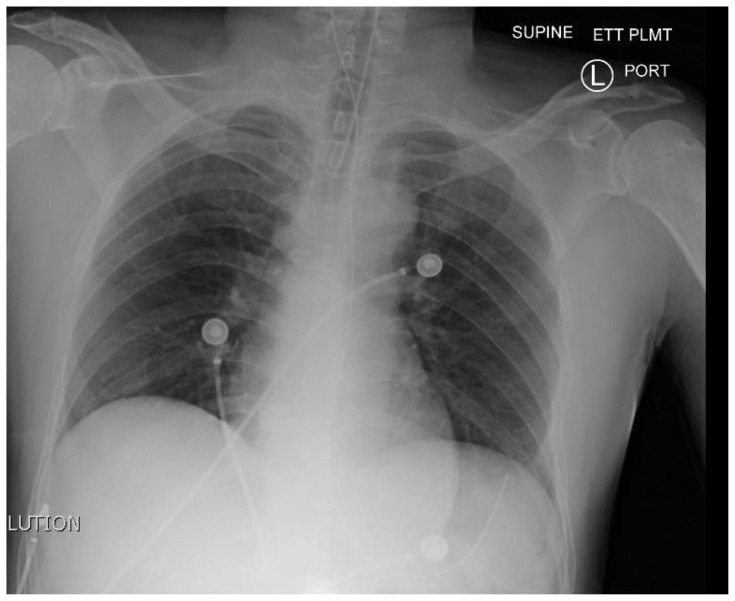


**Figure:**
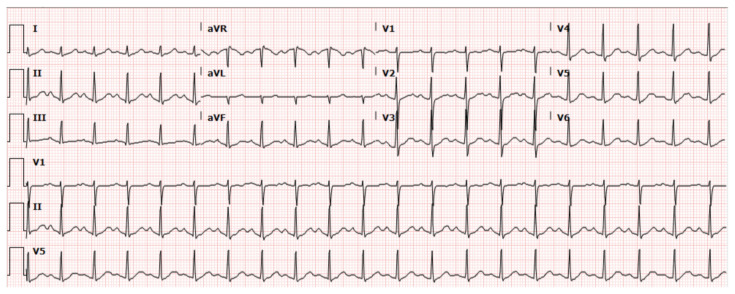


**Figure:**
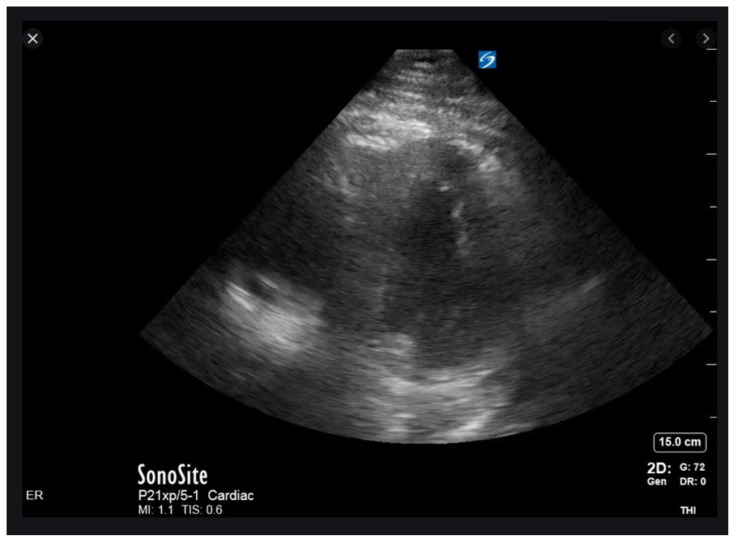


**Figure:**
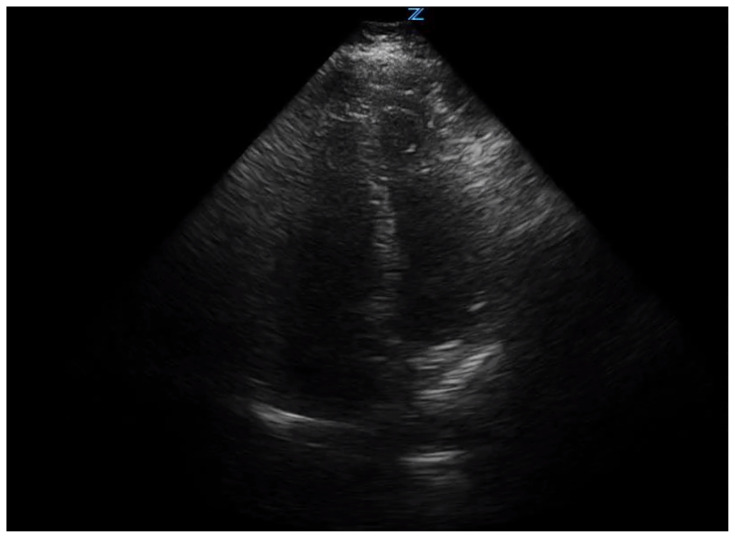


**Table:** SIMULATION EVENTS TABLE:

Minute (state)	Participant action/ trigger	Patient status (simulator response) & operator prompts	Monitor display (vital signs)
**Stage 1**	Initial Assessment	Participants enter the room and find patient actively seizing on stretcher. Initial rapid assessment for airway, breathing and circulation. Need to obtain vital signs. EMS report can be very brief and limited.	T 36.4 °C HR 162 BP 129/57 RR 40 O2 72%
	Suctions Airway	Begin to control airway and suction airway to remove secretions. Oxygen saturation will improve to 76% after suctioning.	O2 76%
	Place on oxygen via non-rebreather mask	Patient continues to seize, and a non-rebreather mask will need to be applied next, if applied oxygen saturation to 83%. If no supplemental oxygen is applied, continue to keep saturation at 76% if suctioned or 72% if no airway management was provided.	O2 83%
	Administer Benzodiazepine	Participant will need to administer either 2 mg of Lorazepam, 5 mg of Versed, or 10 mg of Diazepam to have the seizure successfully stop. Only stop seizing once a benzodiazepine is administered.	HR 133 BP 118/82 RR 36 O2 83%
	Oxygen via Bag Valve Mask Rapid Sequence Intubation ET Tube confirmation Additional history obtained EKG obtained Labs/Arterial Blood Gas (ABG)	After seizure stops, the oxygen saturation should remain at 83%. Participants will need to administer oxygen via a bag valve mask. Respiratory rate should be between 10–14 to have an oxygen saturation improve to 91–93%. The next step is to rapid sequence intubate, using appropriately dosed sedative and paralytics. Medications should be administered in proper order and endotracheal placement confirmed by capnography and auscultation. Chest radiograph with proper placement can be provided if asked. Mother can provide additional information about chest pain and shortness of breath prior to the seizure activity. Can also provide information on new hormonal birth control recently started. If EKG is asked, provide sinus tachycardia EKG. If iSTAT or ABG with electrolytes asked for by participants, provide BMP and ABG results.	O2 91–93%
Two minutes after seizure	No intubation	If participants fail to intubate patient while providing bag valve mask ventilations, the oxygen saturation should start to decrease to a range of 70–74% with proper ventilation at a rate of 10–14 per minute.	O2 70–74%
	IV fluids	If participants choose to administer 500–1000 ml of crystalloid IV fluids, the heart rate will decrease from 133 to 120 beats per minute.	HR 120
After ETT done	HR bradycardic	After successful placement of the endotracheal tube and with proper confirmation, the heart rate should become bradycardic from the tachycardia over 30 minutes, ultimately getting to a rate of 23 beats per minute and the patient will lose pulses.	HR 23, no pulse
**Stage 2**	Pulseless, Starts CPR	Heart rate of 23 continues and participants will need to recognize the patient does not have a pulse. If using waveform capnography capabilities, this should decrease to 5 mmHg until CPR is started. Participants should recheck endotracheal tube placement when CPR is started and begin to go through Hs and Ts in ACLS.	
	Atropine	If participants choose to administer Atropine 0.5 mg to 1 mg, the heart rate can increase to 30, but the patient will still not have a pulse.	HR 30, no pulse
	Additional Atropine	No Changes.	
	Epinephrine Consider Hs/Ts	Participants should be administering epinephrine 1 mg every 3–5 minutes throughout the cardiac arrest. Heart rate can increase to 60 on monitor but no pulse will be obtained. Ensure good quality compressions are being done at a rate of 100–120 with proper depth and recoil. Call for bedside ultrasound while going through Hs and Ts. Provide bedside ultrasound images.	HR 60, no pulse
	Recognition of right heart strain tPA given	Participants recognize right heart strain on ultrasound image and consider thrombolytic therapy. Can consult pharmacy for dose; however pharmacy will inform that this is an off-label use and there is no exact dose recommendation. Pharmacy can tell participants that in this situation for adults, 25–50 mg of tPA is normally administered. Pulse returns 3 minutes after administration (may “jump time” to simulate true onset of tPA).	HR 120 BP 90/60 RR 6 O2 90%
	If no tPA given after 3 rounds of ACLS	Nurse suggests tPA to be given (“What do you think is going on? Is there anything else we could try?” or “Do you think a clot busting medication would work?”)	
**Stage 3**	Post-ROSC	Once ROSC is obtained, no additional changes or medications are needed. Participants should consider contacting an ECMO consultant for the diagnosis of massive PE, and if asked for, provide a consultant. Obtain lab work and provide labs if ordered. May ask for chest x-ray, provide attached chest x-ray.	
Case Completion	Disposition	Call PICU or arrange a proper transfer to PICU or ECMO capable center. Update family on suspected diagnosis and what the next steps are. Simulation ends.	

**Table:** 

**Critical Actions:Stage 1 actions:** 1. Team leader assigns tasks 2. Obtain history from EMS and parent 3. Bag valve mask applied to patient 4. Administer benzodiazepine 5. Place on cardiac monitor 6. Obtain blood glucose level 7. Obtain IV access 8. Intubate patient **Stage 2 actions:** 9. Begin CPR 10. Determine rhythm is PEA 11. Check blood glucose level if not already done 12. Begin ACLS with considerations for PEA 13. Bedside ultrasound 14. Update parent 15. Administer thrombolytics 16. If choosing to do prolonged resuscitation, consider ECMO **Stage 3 actions:** 17. Admit to PICU or arrange transfer 18. Update parent	0:00

**Table:** 

	Milestone	Did not achieve level 1	Level 1	Level 2	Level 3
1	**Emergency Stabilization (PC1)**	□ Did not achieve Level 1	□ Recognizes abnormal vital signs	□ Recognizes an unstable patient, requiring intervention Performs primary assessment Discerns data to formulate a diagnostic impression/plan	□ Manages and prioritizes critical actions in a critically ill patient Reassesses after implementing a stabilizing intervention
2	**Performance of focused history and physical (PC2)**	□ Did not achieve Level 1	□ Performs a reliable, comprehensive history and physical exam	□ Performs and communicates a focused history and physical exam based on chief complaint and urgent issues	□ Prioritizes essential components of history and physical exam given dynamic circumstances
3	**Diagnostic studies (PC3)**	□ Did not achieve Level 1	□ Determines the necessity of diagnostic studies	□ Orders appropriate diagnostic studies. Performs appropriate bedside diagnostic studies/procedures	□ Prioritizes essential testing Interprets results of diagnostic studies Reviews risks, benefits, contraindications, and alternatives to a diagnostic study or procedure
4	**Diagnosis (PC4)**	□ Did not achieve Level 1	□ Considers a list of potential diagnoses	□ Considers an appropriate list of potential diagnosis May or may not make correct diagnosis	□ Makes the appropriate diagnosis Considers other potential diagnoses, avoiding premature closure
5	**Pharmacotherapy (PC5)**	□ Did not achieve Level 1	□ Asks patient for drug allergies	□ Selects an medication for therapeutic intervention, consider potential adverse effects	□ Selects the most appropriate medication and understands mechanism of action, effect, and potential side effects Considers and recognizes drug-drug interactions
6	**Observation and reassessment (PC6)**	□ Did not achieve Level 1	□ Reevaluates patient at least one time during case	□ Reevaluates patient after most therapeutic interventions	□ Consistently evaluates the effectiveness of therapies at appropriate intervals
7	**Disposition (PC7)**	□ Did not achieve Level 1	□ Appropriately selects whether to admit or discharge the patient	□ Appropriately selects whether to admit or discharge Involves the expertise of some of the appropriate specialists	□ Educates the patient appropriately about their disposition Assigns patient to an appropriate level of care (ICU/Tele/Floor) Involves expertise of all appropriate specialists
9	**General Approach to Procedures (PC9)**	□ Did not achieve Level 1	□ Identifies pertinent anatomy and physiology for a procedure Uses appropriate Universal Precautions	□ Obtains informed consent Knows indications, contraindications, anatomic landmarks, equipment, anesthetic and procedural technique, and potential complications for common ED procedures	□ Determines a back-up strategy if initial attempts are unsuccessful Correctly interprets results of diagnostic procedure
20	**Professional Values (PROF1)**	□ Did not achieve Level 1	□ Demonstrates caring, honest behavior	□ Exhibits compassion, respect, sensitivity and responsiveness	□ Develops alternative care plans when patients’ personal beliefs and decisions preclude standard care
22	**Patient centered communication (ICS1)**	□ Did not achieve level 1	□ Establishes rapport and demonstrates empathy to patient (and family) Listens effectively	□ Elicits patient’s reason for seeking health care	□ Manages patient expectations in a manner that minimizes potential for stress, conflict, and misunderstanding. Effectively communicates with vulnerable populations, (at risk patients and families)
23	**Team management (ICS2)**	□ Did not achieve level 1	□ Recognizes other members of the patient care team during case (nurse, techs)	□ Communicates pertinent information to other healthcare colleagues	□ Communicates a clear, succinct, and appropriate handoff with specialists and other colleagues Communicates effectively with ancillary staff

## INSTRUCTOR MATERIALS

**Case Title:** Cardiac Arrest in an Adolescent with Pulmonary Embolism

**Case Description & Diagnosis (short synopsis):** A 13-year-old female presents with seizure due to hypoxia. After initial resuscitation, she goes into PEA arrest due to pulmonary embolism. The case has three stages: 1) Managing seizure followed by intubation, 2) PEA cardiac arrest with need to recognize pulmonary embolism and treat with tPA +/− ECMO, 3) post-ROSC care and disposition.

**Equipment or Props Needed:**

High-fidelity adolescent simulator
IV pole
Crash cart
Phone
Airway equipment.

Of note, this case could also be run using a low fidelity mannikin with task trainers for intubation and CPR. Our mannikin does not have ultrasound capabilities, and we had our learners describe how they would perform the ultrasound to get the required images.

**Confederates needed:**

A nurse, a parent, one person to answer phone as the consultant/pharmacy.

**Stimulus Inventory:**

**Background and brief information:** A 13-year-old female presents via EMS after a seizure at home after complaining of chest pain, shortness of breath, and not feeling well earlier in the day. Arrives seizing in the ED, accompanied by EMS and a parent. She has no history of seizures or recent illness.

**Initial presentation:** Presents via EMS, seizing on arrival.

**How the scene unfolds:** There are 3 stages to this simulation. Stage 1: The patient arrives seizing and will require anti-epileptics. She remains hypoxic and will need to be intubated using RSI. After intubation, she becomes bradycardic and goes into a PEA arrest, which starts Stage 2. Stage 2: PEA arrest requiring CPR and appropriate ACLS. Bedside ultrasound will assist in diagnosis of PE, and thrombolytics should be administered to achieve ROSC, and begin Stage 3. Stage 3: Patient stabilization and disposition with discussions with family members and admitting team.

**Critical actions:**

**Stage 1 actions:**

1. Team leader assigns tasks
2. Obtain history from EMS and parent
3. Bag valve mask applied to patient
4. Administer benzodiazepine
5. Place on cardiac monitor
6. Obtain blood glucose level
7. Obtain IV access
8. Intubate patient

**Stage 2 actions:**

9. Begin CPR
10. Determine rhythm is PEA
11. Check blood glucose level if not already done
12. Begin ACLS with considerations for PEA
13. Bedside ultrasound
14. Update parent
15. Administer thrombolytics
16. If choosing to do prolonged resuscitation, consider ECMO

**Stage 3 actions:**

17. Admit to PICU or arrange transfer
18. Update parent

**Case Title:** Cardiac Arrest in an Adolescent with Pulmonary Embolism

**Chief Complaint:** Seizures / Altered Mental Status

**EMS Report (if asked):** Called for a sick person. She did not appear well. Shortly after arrival she had tonic-clonic seizure which self-resolved after two minutes. Since then, she occasionally moans and withdraws from painful stimuli. She begins seizing again upon arrival at the Emergency Department

**General Appearance:** Actively seizing.

**Primary Survey:**

- **Airway:** Some secretions in airway
- **Breathing:** Shallow and tachypneic
- **Circulation:** Central and peripheral pulses present, tachycardic

**History:**

- **History of present illness:** “Mandy” is a 13-year-old female who presents via EMS actively seizing. A parent is also present. She states that Mandy was feeling sick earlier in the day, reporting chest pain across her chest, and shortness of breath. She was sitting on a couch with her parent and became increasingly unresponsive, so 911 was called. She has been previously healthy.
- **Past medical history:** metromenorrhagia and resulting anemia since menses at age 11
- **Past surgical history:** None
- **Medications:** Drospirenone-Ethinyl Estradiol 1 tab daily, Iron Sulfate 325mg daily
- **Allergies:** None
- **Social history:** No tobacco, alcohol or drugs, lives at home with parents, not sexually active
- **Family history:** None

**Secondary Survey/Physical Examination:**

- **General appearance:** After seizure abates, she is unresponsive to all stimuli, shallow respirations with tachypnea.
- **HEENT:**
  ○ **Head:** Normal
  ○ **Eyes:** 4 mm, reactive B/L
  ○ **Ears:** Normal
  ○ **Nose:** Normal
  ○ **Throat:** Secretions present
- **Neck:** Normal, no crepitus
- **Heart:** Tachycardic, no murmur
- **Lungs:** Shallow, tachypnea, clear lungs
- **Abdominal/GI:** Normal
- **Genitourinary:** Normal
- **Rectal:** Normal
- **Extremities:** Normal
- **Back:** Normal
- **Neuro:** Glasgow Coma Scale 3, unresponsive to all stimuli, has gag reflex
- **Skin:** some pallor, otherwise normal
- **Lymph:** Normal
- **Psych:** Unable to assess

**Stage 2:**

**Updated Information:** PEA arrest, underlying rhythm is narrow complex bradycardia.

**Primary Survey:**

- **Airway:** Pt should be intubated in stage 1. If simulator unable to be intubated, have the learner describe the steps of RSI
- **Breathing:** Absent, lung sounds present with BVM
- **Circulation:** Absent Pulses

**Stage 3:**

**Updated Information:** ROSC obtained

**Primary Survey:**

- **Airway:** Secured with Endotracheal Tube
- **Breathing:** Patient breaths 6 times a minute on own, clear lungs
- **Circulation:** Central and Peripheral Pulses present

**Physical Exam Changes:** (Same as Stage 1 unless noted below)

- Lungs: Patient rate at 6, lungs clear
- Neuro: Withdrawal from painful stimuli, no eye opening

**Results:**

*Chest Radiograph (CXR)*

Normal CXR and Post-Intubation CXR.

Image source: JETem. https://jetem.org/ettcxr/. CC BY 4.0.

*EKG Sinus Tachycardia ECG:*

Sinus Tachycardia.

Image source: DDx of. https://ddxof.com/sinus-tachycardia/ecg_5/. CC BY-SA 4.0.

*Ultrasound showing right heart strain, Parasternal Long:*

Image source: Mead, T. The D-sign: Right Ventricular Strain. The POCUS Atlas.

https://www.thepocusatlas.com/new-blog/d-sign. CC BY-NC 4.0

*Ultrasound image showing right heart strain, Apical 4 chamber*

Image source: Image with permissions from: Kathleen Fitzgibbon, MD, UPMC Harrisburg, Harrisburg, Pa.

## OPERATOR MATERIALS

**Diagnosis:**

Massive Pulmonary Embolism

**Disposition:**

PICU or transfer to a center with PICU capabilities

## DEBRIEFING AND EVALUATION PEARLS

### Massive Pulmonary Embolism

- Background: Pulmonary Embolism (PE) is rare in children. Children with PE can present with symptoms including chest pain, tachypnea, cough, tachycardia, dyspnea, or sudden collapse. As clot burden increases in massive PE, blood is no longer able to be transported to the lungs from the right side of the heart, leading to right heart strain and eventually right ventricular failure.^4^ This process leads to fatality due to hypoxemia and obstructive shock.
- Etiology: Incidence of venous thromboembolism (VTE) in children is 0.14 to 0.21 per 10,000 children in the general population. Pulmonary embolism accounts for about 15% of these cases in children.^6^ Risk factors for pulmonary embolism in children include central lines, trauma, immobility, estrogen-containing contraceptives, inflammatory conditions, malignancy, heart disease, dehydration, and obesity.^5^
- Diagnostic Testing: Unfortunately, D-dimer and Wells scoring tools appear to lack utility when applied to children.^5^ The gold standard for testing is a CT scan with contrast assessing for pulmonary embolism. In the incident of an acutely unstable child where it is unsafe for CT imaging, bedside ultrasound can help to identify right heart strain.
- Treatment Options: In massive pulmonary embolism, thrombolytic agents can be administered in children and should be used in hemodynamically unstable patients. However, there is currently no FDA approved pediatric bolus dose of alteplase or other thrombolytic drugs. Administration of thrombolytics should be done cautiously, and if possible, with consultation of a pediatric hematologist.^7^ Use of ECMO should also be considered when patients are in a peri-arrest or cardiac arrest state if the patient is being resuscitated in a facility capable of emergently performing the procedure. Other treatment options for massive PE include catheter directed thrombolytics, catheter thrombectomy, and open surgical thrombectomy in more stable patients. Early consultation for these procedures in massive and submassive pulmonary embolism has potential to decrease mortality.^11^

### Estrogen Containing Oral Contraceptives

- Women taking estrogen-containing oral contraceptives (OCPs) have a three to five times higher risk of thromboembolism when compared to the general population.^8^ The risk of VTE is highest within the first few months of starting the medication. Prior to initiating estrogen-containing OCPs, prescribers should note other patient risk factors such as smoking, obesity, and polycystic ovary syndrome (PCOS). Alternative forms of contraception should be recommended in patients with multiple risk factors.^9^,^10^
- Overall, use of estrogen containing OCPs is still very safe in the general population. Risk stratification should be used when a gynecologist or primary provider engages in contraceptive counseling.

**Other debriefing points:**

If learners did not obtain medication history, consider asking, “Would there have been a benefit to getting a full medical history from the parent? When can this happen during a busy resuscitation?”

If learners did not suggest ultrasound, consider asking, “Were there any other bedside tests that could have helped make the correct diagnosis?”

## SIMULATION ASSESSMENT

*Cardiac Arrest in an Adolescent with Pulmonary Embolism*

Learner: _________________________________________

***Assessment Timeline***

This timeline is to help observers assess their learners. It allows observer to make notes on when learners performed various tasks, which can help guide debriefing discussion.

**Critical Actions:**

**Stage 1 actions:**

□ Team leader assigns tasks
□ Obtain history from EMS and parent
□ Bag valve mask applied to patient
□ Administer benzodiazepine
□ Place on cardiac monitor
□ Obtain blood glucose level
□ Obtain IV access
□ Intubate patient

**Stage 2 actions:**

□ Begin CPR
□ Determine rhythm is PEA
□ Check blood glucose level if not already done
□ Begin ACLS with considerations for PEA
□ Bedside ultrasound
□ Update parent
□ Administer thrombolytics
□ If choosing to do prolonged resuscitation, consider ECMO

**Stage 3 actions:**

□ Admit to PICU or arrange transfer
□ Update parent

**Summative and formative comments:**

**Milestones assessment:**
