# Supplementary material for: Cardiac Arrest in an Adolescent with Pulmonary Embolism
Source: J Educ Teach Emerg Med. 2021 Oct 15;6(4):S112–37. doi: 10.21980/J8135T (PMC10332741; doi:10.21980/J8135T)
Supplement: Supplementary file 1 [file JETem-6-4-S112-supp1.pptx]

## Slide 1
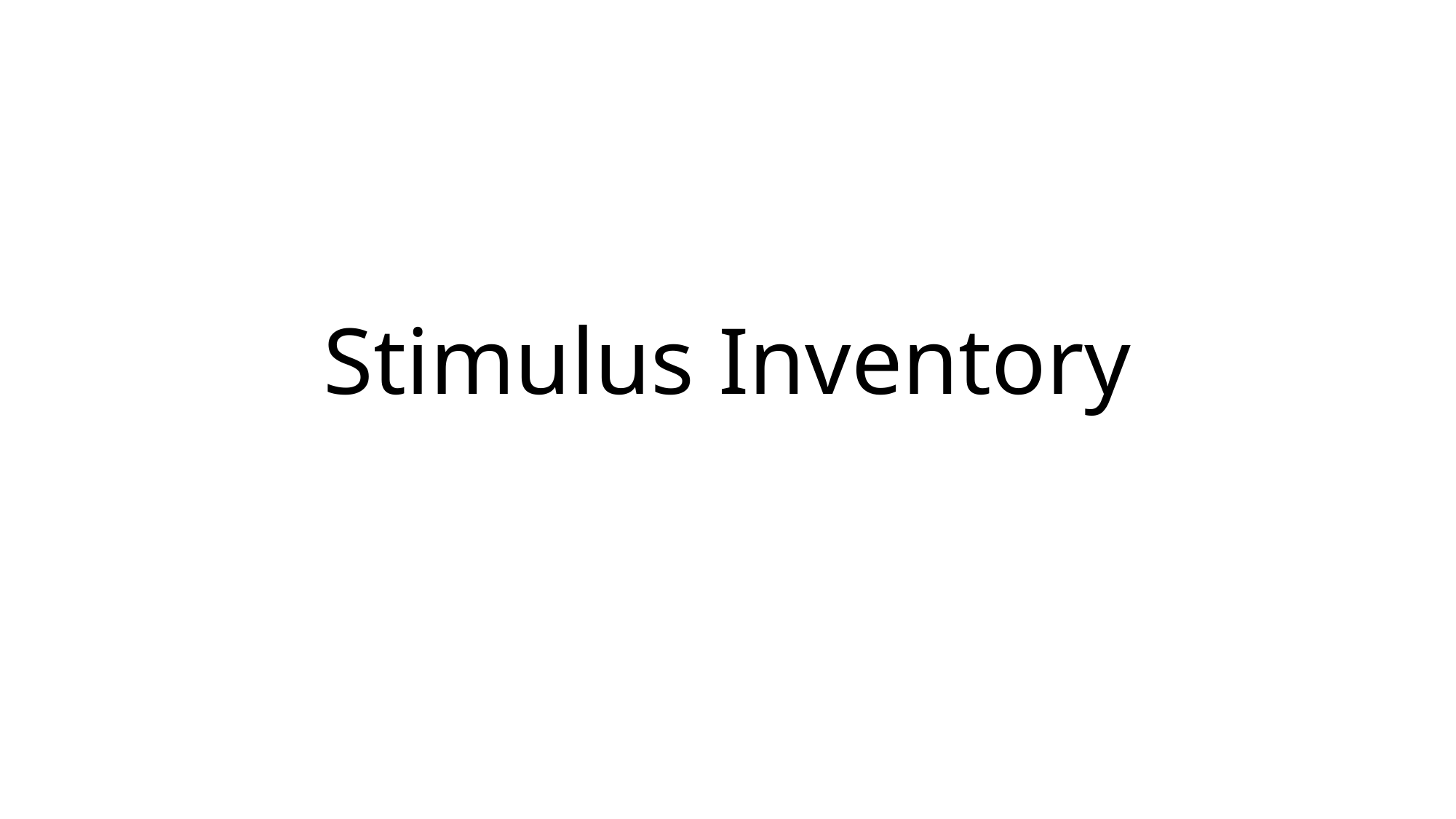

# Stimulus Inventory

## Slide 2
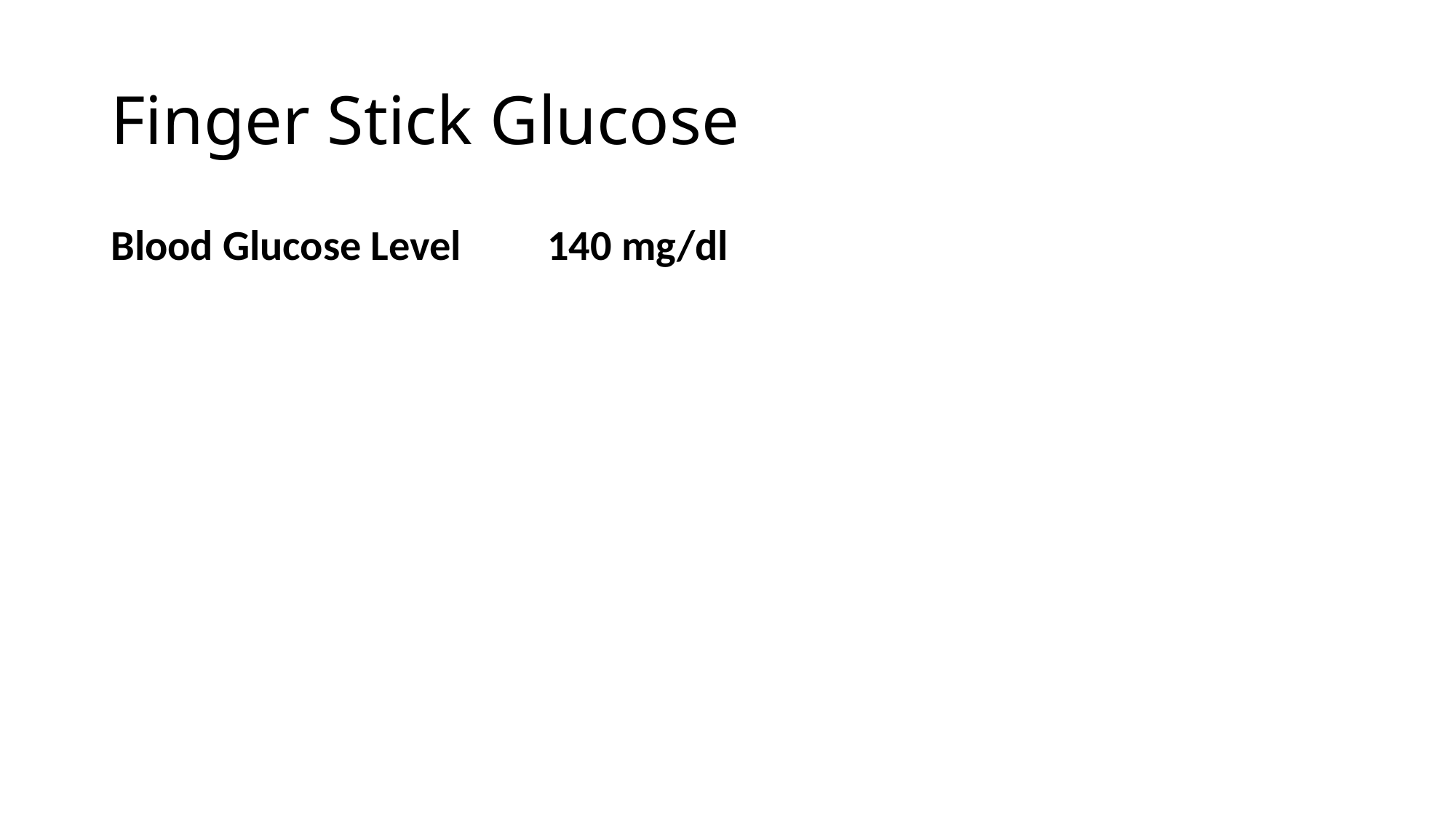

# Finger Stick Glucose
Blood Glucose Level 	140 mg/dl

## Slide 3
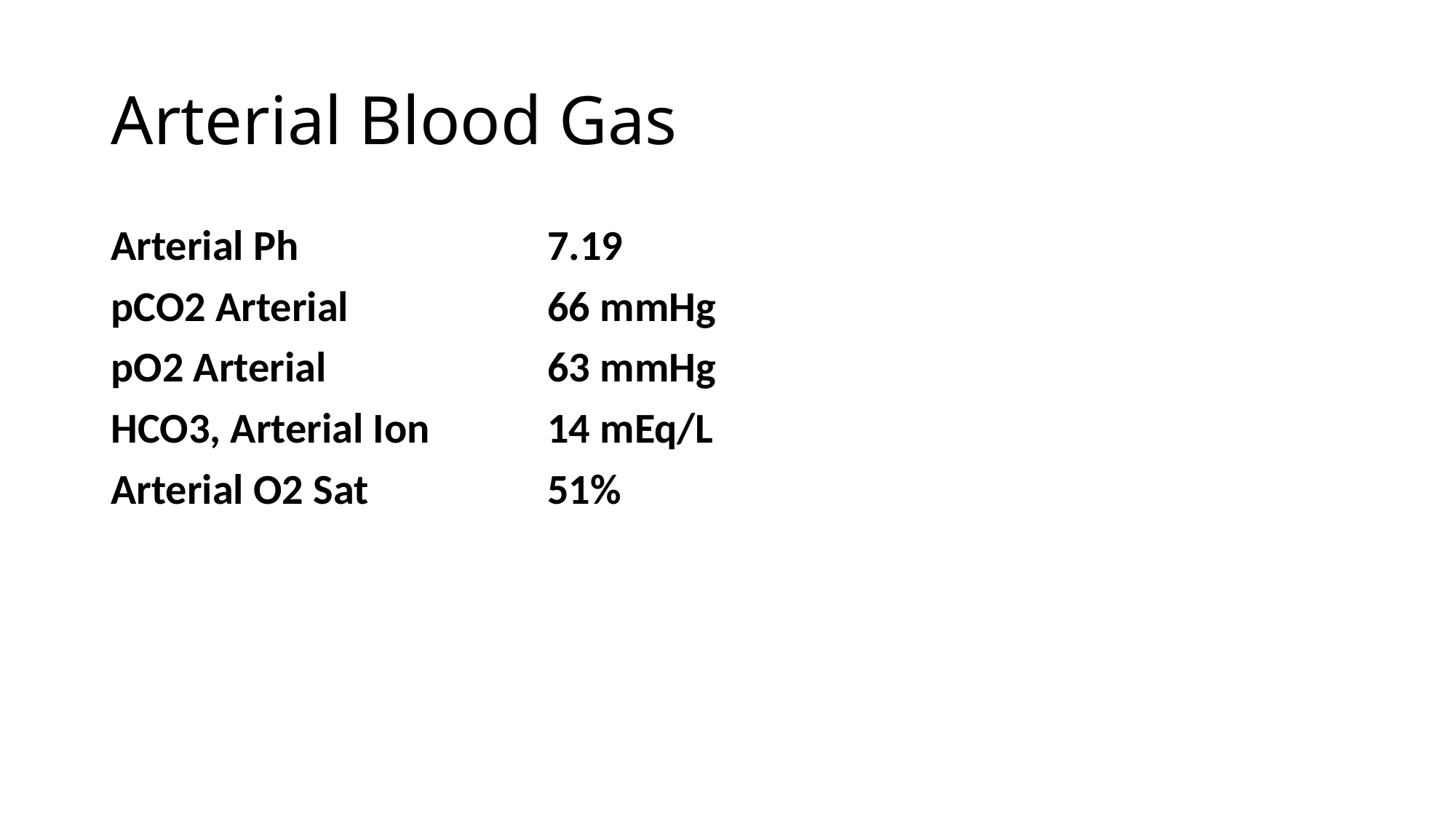

# Arterial Blood Gas
Arterial Ph			7.19
pCO2 Arterial		66 mmHg
pO2 Arterial			63 mmHg
HCO3, Arterial Ion		14 mEq/L
Arterial O2 Sat 		51%

## Slide 4
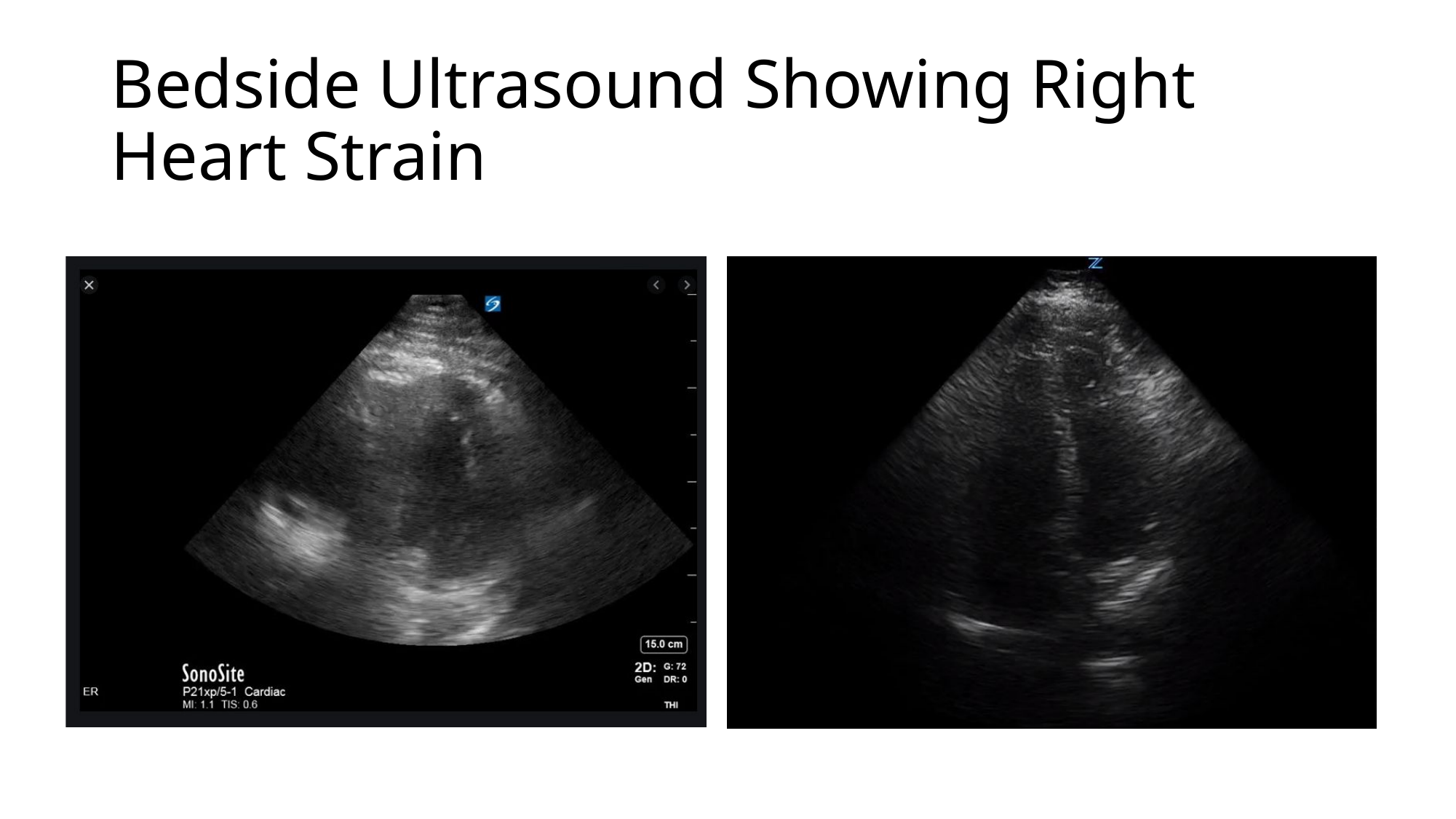

# Bedside Ultrasound Showing Right Heart Strain

## Slide 5
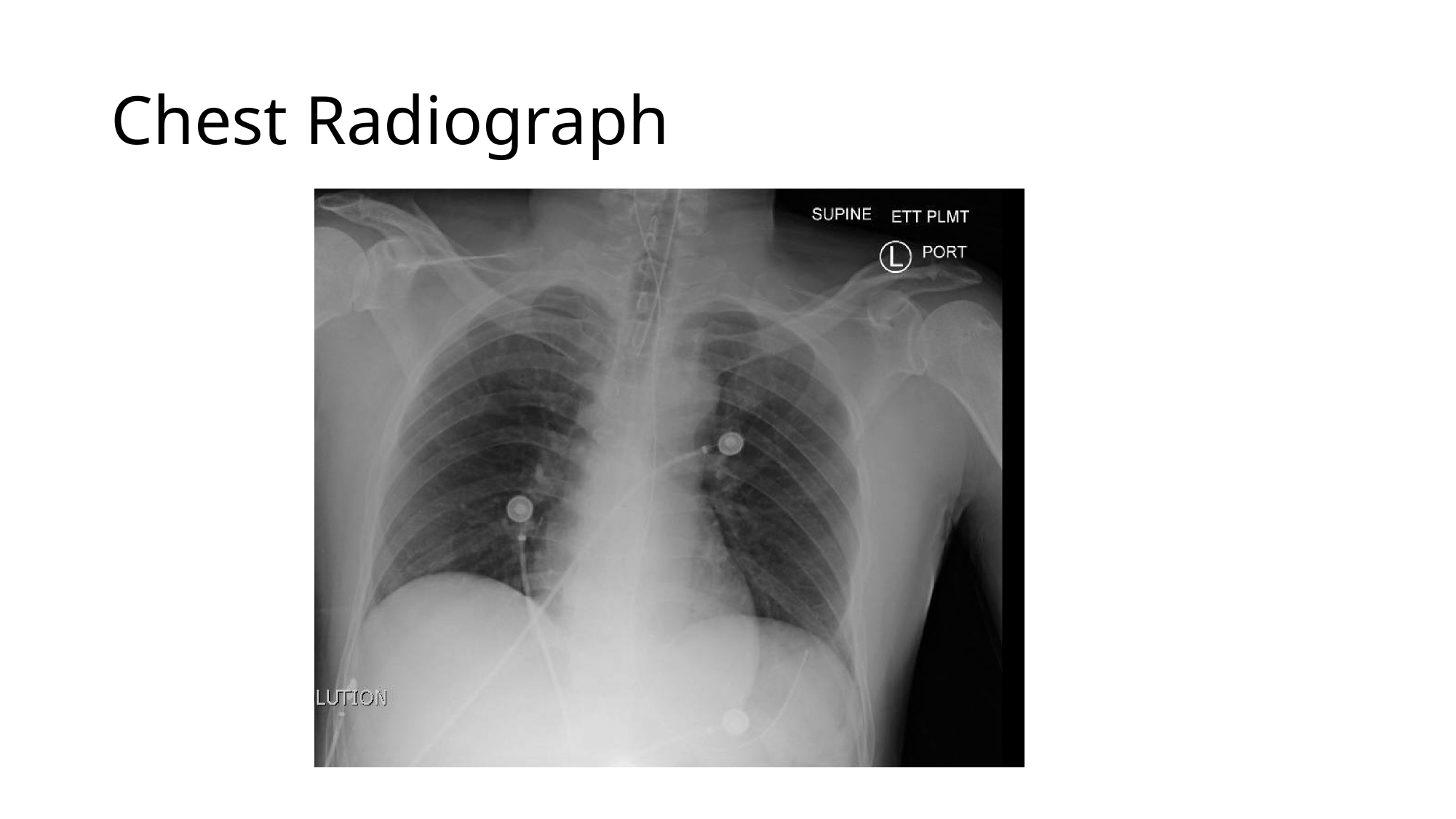

# Chest Radiograph

## Slide 6
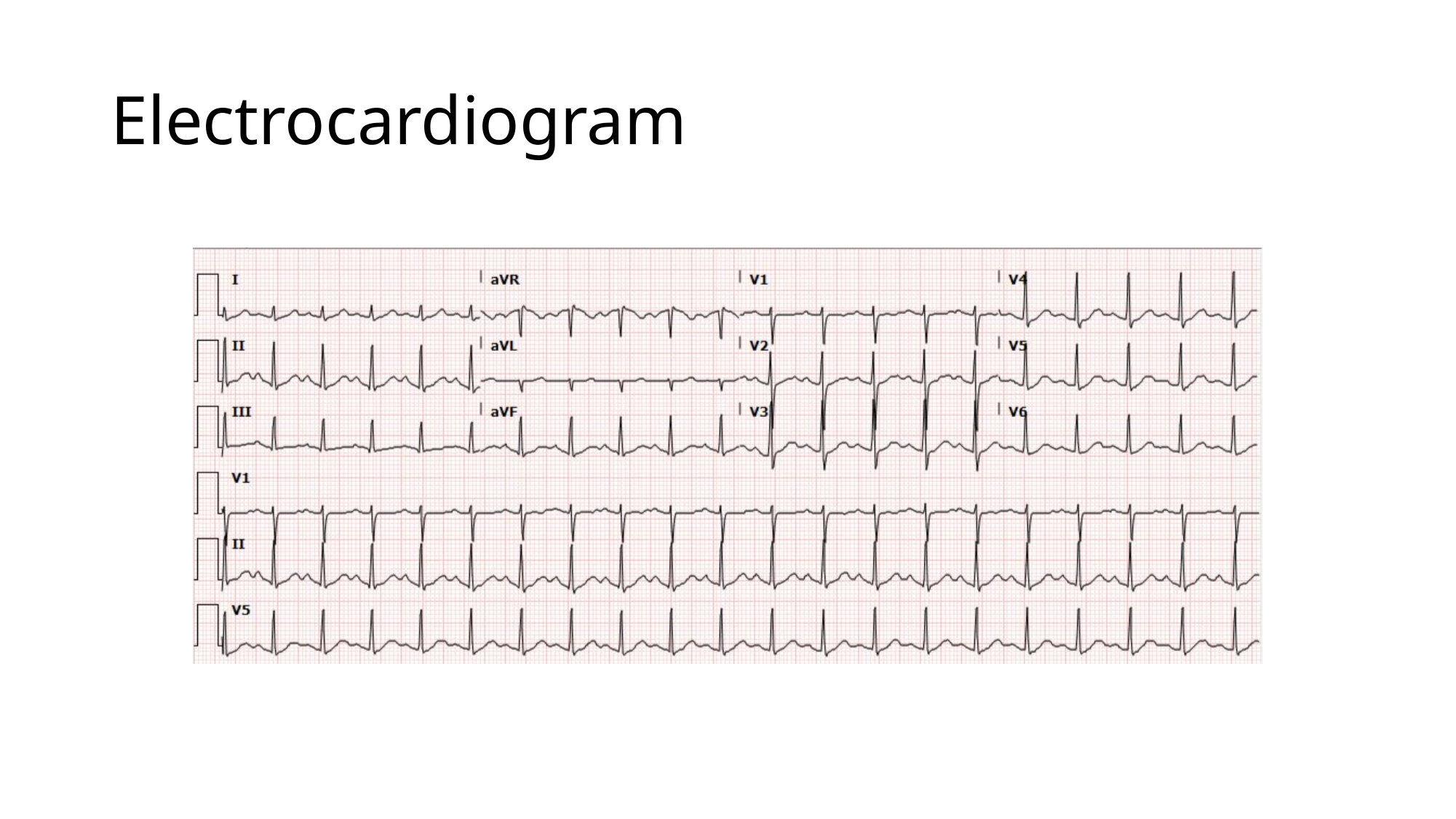

# Electrocardiogram

## Slide 7
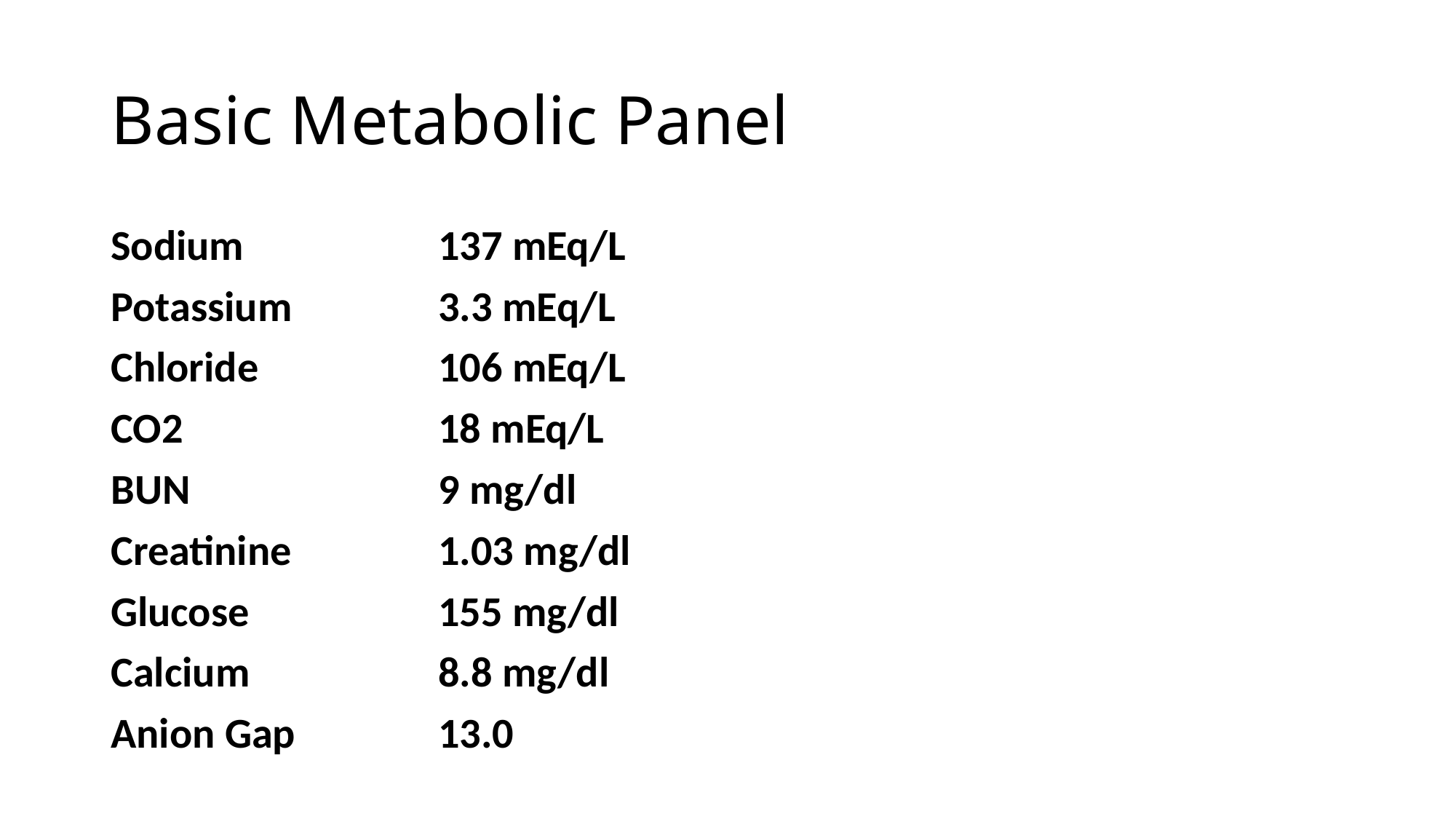

# Basic Metabolic Panel
Sodium		137 mEq/L
Potassium		3.3 mEq/L
Chloride		106 mEq/L
CO2			18 mEq/L
BUN			9 mg/dl
Creatinine		1.03 mg/dl
Glucose		155 mg/dl
Calcium		8.8 mg/dl
Anion Gap		13.0

## Slide 8
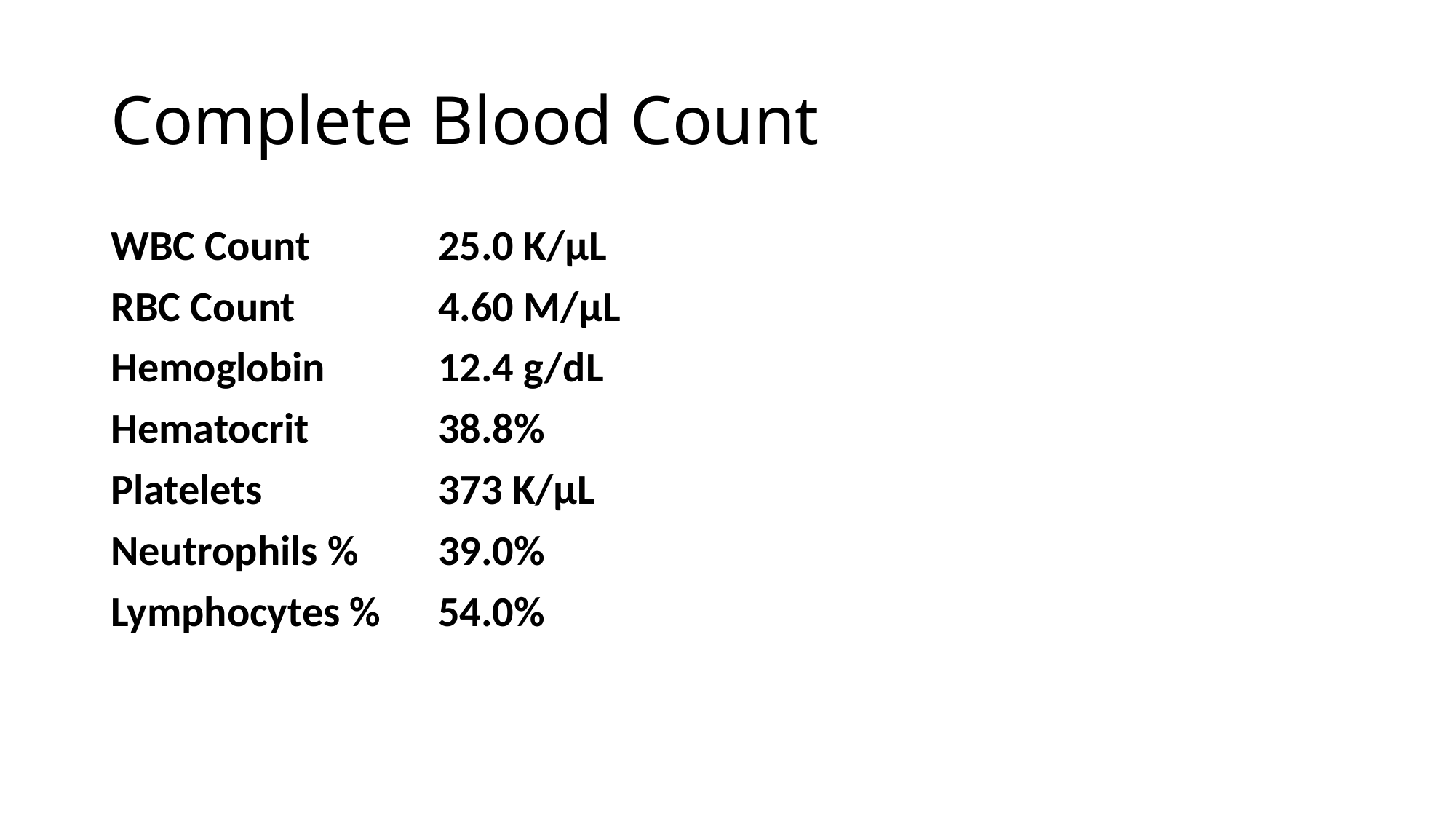

# Complete Blood Count
WBC Count		25.0 K/µL
RBC Count		4.60 M/µL
Hemoglobin		12.4 g/dL
Hematocrit		38.8%
Platelets		373 K/µL
Neutrophils %	39.0%
Lymphocytes %	54.0%

## Slide 9
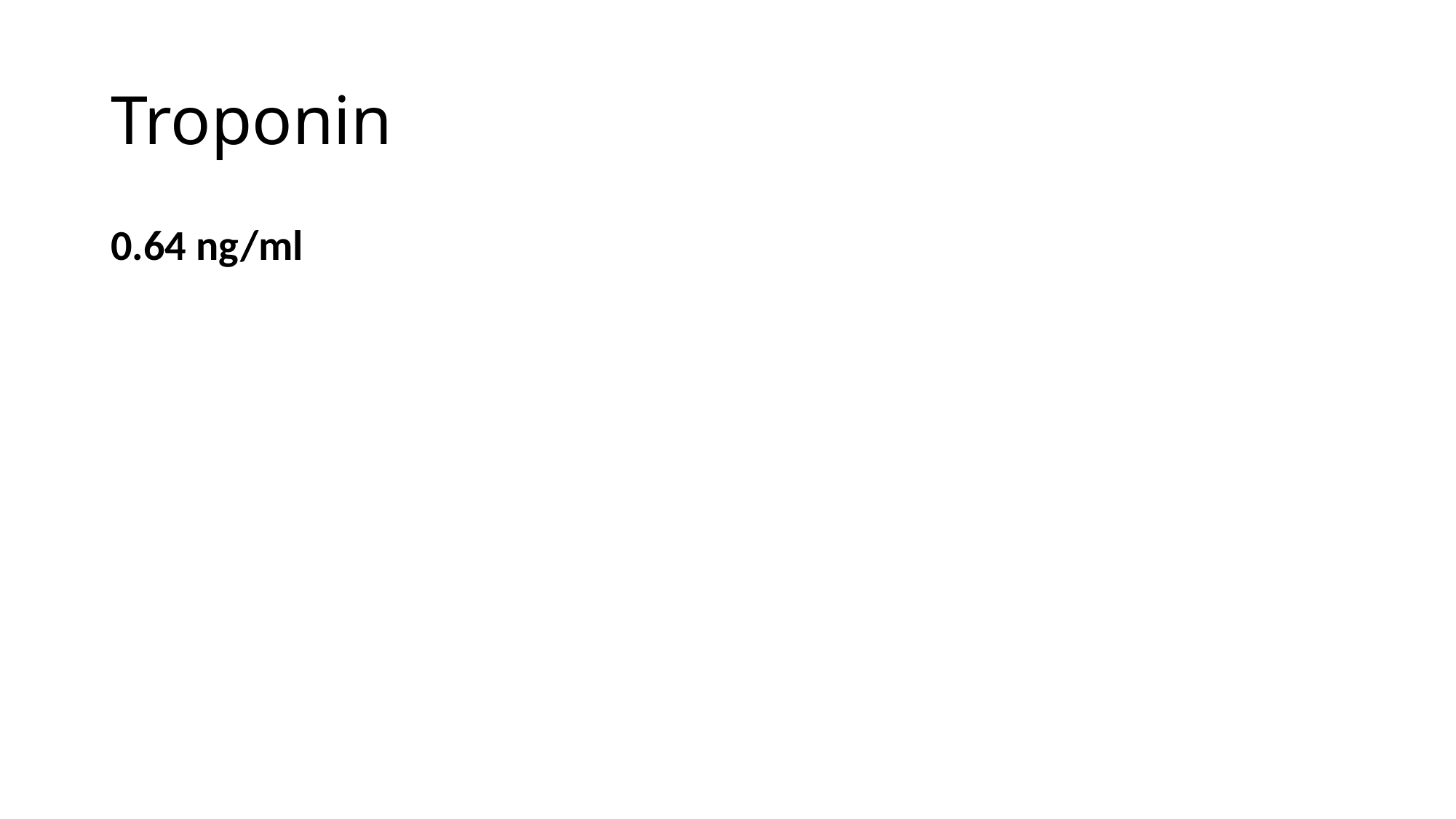

# Troponin
0.64 ng/ml

## Slide 10
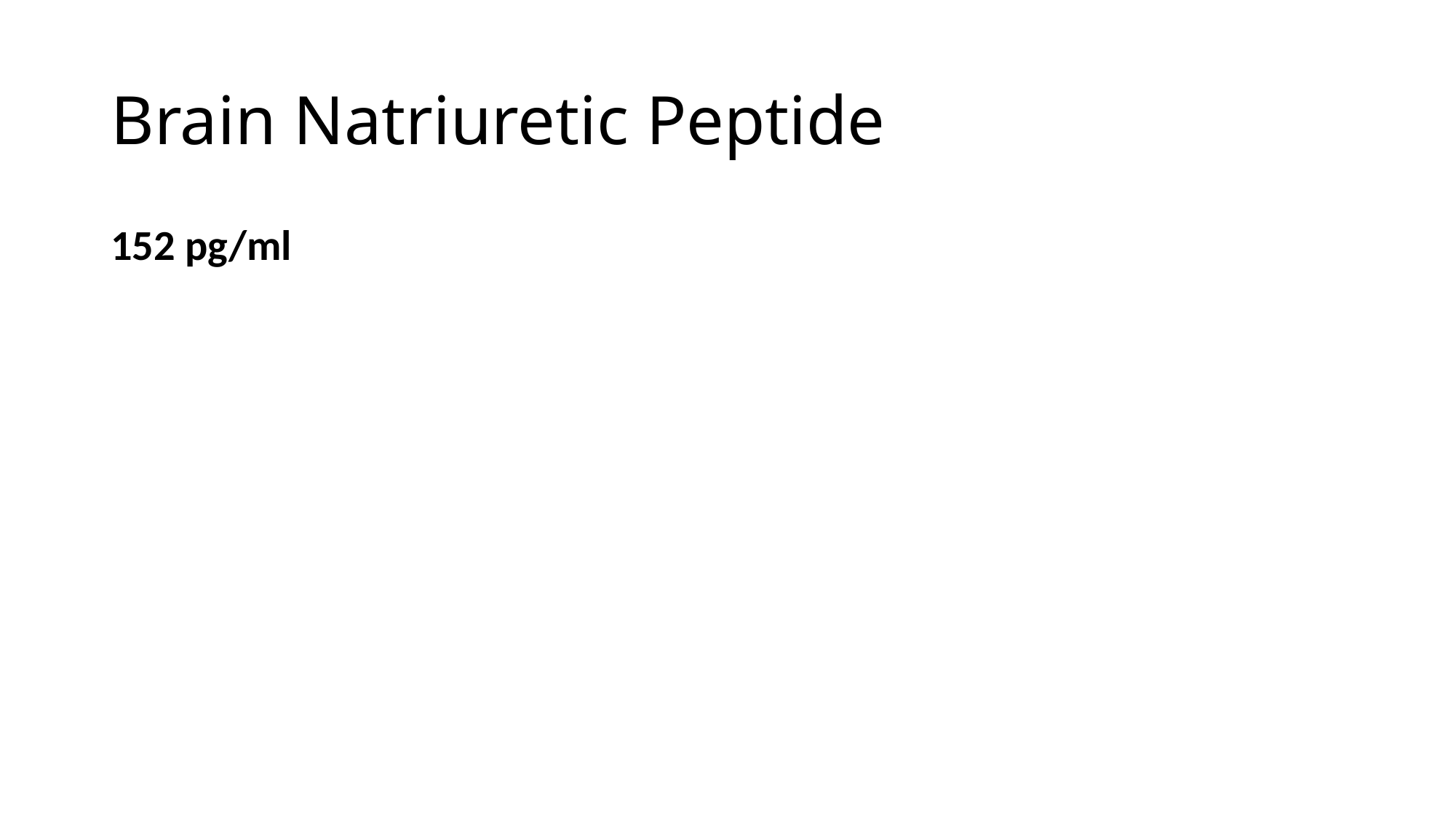

# Brain Natriuretic Peptide
152 pg/ml

## Slide 11
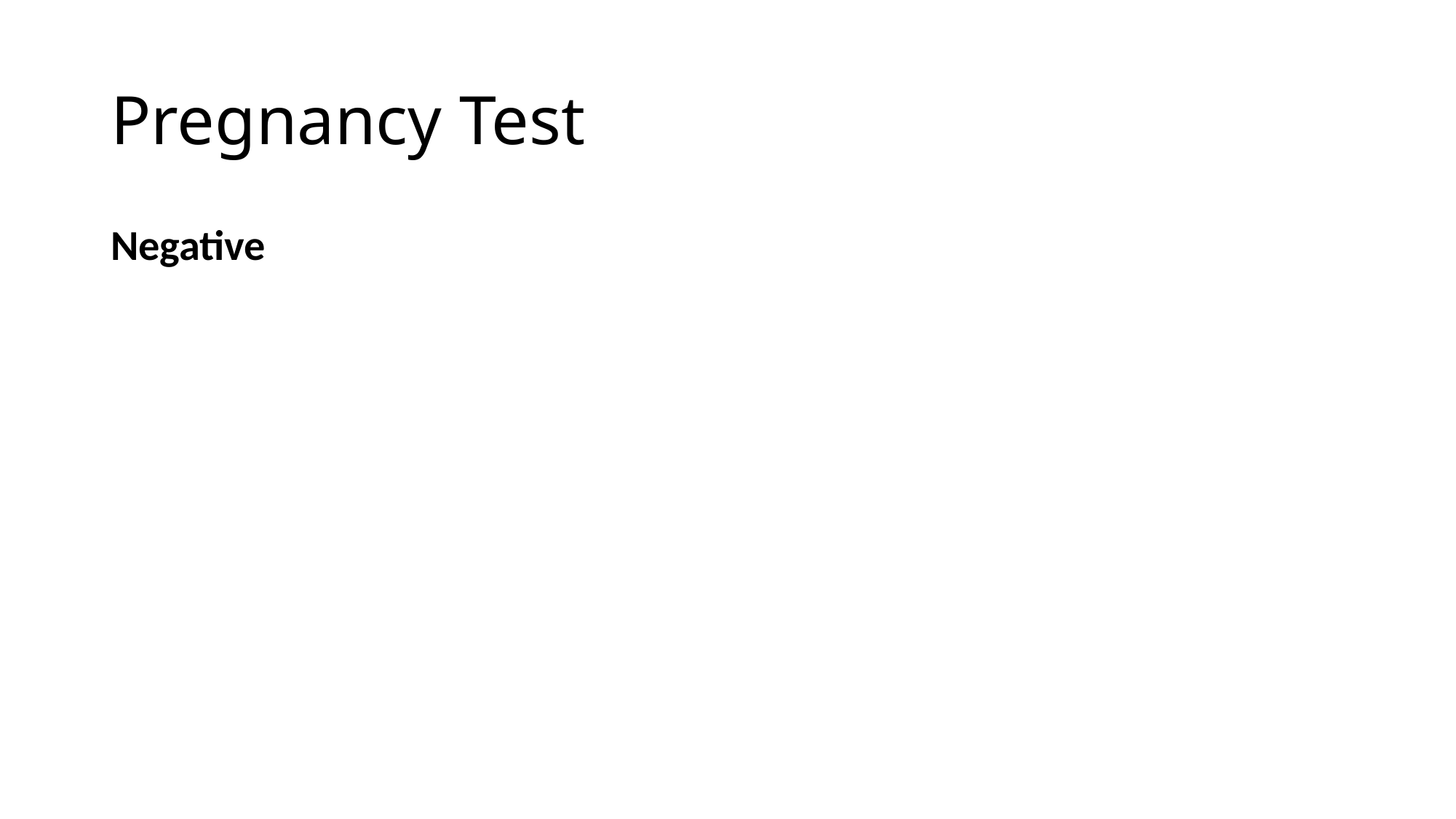

# Pregnancy Test
Negative
